# Associations of sedentary behaviour and physical activity with stress-related sleep disturbance among adolescents in 69 countries: a population-based study

**DOI:** 10.7189/jogh.16.04049

**Published:** 2026-02-06

**Authors:** Jingpeng Li, Yongliang Zhu, Danyi Huang, Mengna Pan, Fei Li, Liuqing Li, Jiahong Sun, Chuanwei Ma, Bingsong Zhang

**Affiliations:** 1 Department of Epidemiology and Health Statistics, The First Dongguan Affiliated Hospital, School of Public Health, Guangdong Medical University, Dongguan, China; 2 Department of children and wellness, The First Dongguan Affiliated Hospital, Guangdong Medical University, Dongguan, China; 3 Department of Preventive Medicine, The First Dongguan Affiliated Hospital, School of Public Health, Guangdong Medical University, Dongguan, China

## Abstract

**Background:**

Stress-related sleep disturbance have become a serious public health problem among adolescents worldwide. There is a paucity of research employing standardised methodologies to evaluate the association between sedentary behaviour, physical activity, and stress-related sleep disturbance among adolescents. We aimed to examine the association between sedentary behaviour and/or physical activity with stress-related sleep disturbance among adolescents worldwide.

**Methods:**

We used data from the Global School-based Student Health Survey (GSHS) conducted from 2010 to 2019, encompassing 275 483 adolescents aged 12–17 years across 69 countries. Multi-variable logistic regression analysis was performed to assess the independent and joint effect of sedentary behaviour and physical activity on stress-related sleep disturbance.

**Results:**

30.0% of adolescents spend ≥2 hours in sedentary behaviour daily, only 15.4% engage in physical activity for over one hour each day, and 8.3% of adolescents suffer from stress-related sleep disturbances. Compared with those who engaged sedentary behaviour <2 hours/day, 3–4 hours (odds ratio (OR) = 1.168; 95% CI = 1.027–1.329), 5–6 hours (OR = 1.413; 95% CI = 1.169–1.707), and ≥7 hours (OR = 1.792; 1.548–2.076) of daily sedentary behaviour are positively associated with stress-related sleep disturbances among adolescents. Compared with adolescents with low sedentary behaviour and sufficient physical activity, adolescents with low sedentary behaviour and insufficient physical activity (OR = 1.303; 95% CI = 1.052–1.615), high sedentary behaviour and insufficient physical activity (OR = 1.666; 95% CI = 1.350–2.056), and adolescents with high sedentary behaviour and sufficient physical activity (OR = 1.852; 95% CI = 1.432–2.396) are positively associated with stress-related sleep disturbance.

**Conclusions:**

Sedentary behaviour is associated with a higher risk of stress-related sleep disturbances among adolescents. Reducing sedentary behaviour time may serve as a potential intervention strategy for addressing stress-related sleep disturbances, while the potential benefits of increasing physical activity require further research and validation.

Sleep disturbance represents a significant public health concern, impacting 20–50% of the global population [1]. A study conducted in the USA revealed an alarming rise in both medical consultations and the prescription of sleep medications for sleep-related issues between 1999 and 2010 [2]. Adolescence is a critical period of growth and development. Previous research suggests that sleep issues among adolescents tend to intensify over time, manifesting in both internalising and externalising behaviours, and are exacerbated with increasing grade level [3]. Sleep disturbance is reportedly more prevalent in adolescents compared to middle-aged and older adults [4], which may further exacerbate the global burden of sleep disturbance among this demographic. Based on data from the Global School-based Student Health Survey (GSHS) conducted in 82 countries between 2003 and 2015, the pooled prevalence of stress-related sleep disturbance among adolescents aged 12 to 17 years was 9.0% [5]. Therefore, it is imperative to identify the factors associated with stress-related sleep disturbance among adolescents.

Given the high prevalence and burden of sleep disturbance, there is an urgent need for new interventions to help people prevent and reduce sleep problems. An increasing number of studies indicate that there is a correlation between sedentary behaviour and physical activity with sleep disturbance. Based on the National Health and Nutrition Examination Survey database in the USA, Li et al. found that among individuals aged 18 and older, the risk of sleep disturbance increased with increased sedentary time [6]. Boyle et al. also found that sedentary behaviour can increase the risk of insomnia symptoms [7]. In addition, a meta-analysis identified that sedentary behaviour was associated with an increased risk of sleep disturbance [8]. Physical activity can mitigate the harmful effects of sedentary behaviour, such as the risk of death [9]. A study showed that achieving the internationally recommended minimum level of physical activity is effective in reducing the severity of insomnia, such as mood, fatigue and daytime sleepiness [10]. A meta-analysis showed that physical activity improves sleep quality and reduces sleep latency [11]. However, previous studies have mostly focused on adult populations, with few studies assessing the association between sedentary behaviour and physical activity in adolescents, as well as their combined effects on sleep disturbance, using a unified measurement tool [12–14].

Therefore, to address this gap, we hypothesise a ‘dose-response’ relationship between sedentary time, physical activity, and stress-related sleep disturbance in adolescents. Specifically, we propose that greater sedentary time and lower physical activity are each associated with an increased risk of such disturbance.

## METHODS

We obtained the most recent publicly available data from the GSHS surveys conducted between 2010 and 2019 in 69 countries among adolescents aged 12–17 years. The GSHS is an ongoing, school-based, cross-sectional survey that uses the same research methodology, validated questionnaires, and a standardised two-stage cluster sampling strategy in all included countries. The GSHS was developed by the World Health Organization (WHO) and the US Centers for Disease Control and Prevention (CDC). In the first stage, schools were randomly chosen with probability of proportional to their size. In the second stage, classes were randomly chosen from the selected schools, and all students in the selected classes were qualified to take part in the survey. Adolescents in all countries participated voluntarily and no identifiable information was collected. The questionnaire was translated into the local language of each country and then back-translated into English to ensure accuracy. All answers were anonymous. All GSHS surveys in each country obtained approval from either the Health Research Ethics Committee or Ministry of Education, or both. In addition, they obtained informed verbal or written consent from all students’ parents and/or their guardians.

### Definition of sedentary behaviour, physical activity, and stress-related sleep disturbance

The question for assessing sedentary behaviour was: ‘How much time do you spend on a typical or usual day (except for in school or doing homework) sitting and watching television, playing computer games, talking with friends, or doing other sitting activities?’. The corresponding answers were ‘less than 1 hour per day’, ‘1 to 2 hours per day’, ‘3 to 4 hours per day’, ‘5 to 6 hours per day’, ‘7 to 8 hours per day’, and ‘more than 8 hours per day’. In our study, based on the Canadian 24-Hour Movement Guidelines for Children and Youth aged 5–17 years, which recommend limiting screen or sitting time to no more than two hours per day [15]. We defined high sedentary behaviour in our study as engaging in seated activities for ≥2 hours per day, excluding time spent sitting in school settings or completing homework.

The question used to assess physical activity was: ‘During the past seven days, on how many days were you physically active for a total of at least 60 minutes per day?’ (add up all the time you spent on any type of physical activity each day). The answer options available to participants included ‘0 days’, ‘1 day’, ‘2 days’, ‘3 days’, ‘4 days’, ‘5 days’, ‘6 days’, and ‘7 days’. Physical activity was defined as any activity that raises a student’s heart rate and leaves the student out of breath at some point, including leisure time sports, playing sports with friends or walking to school. In our analysis, sufficient physical activity was defined as meeting the WHO recommendation of ≥ 60 minute/day of moderate to vigorous intensity physical activity of any kind during the past seven days.

To assess stress-related sleep disturbance, adolescents were asked: ‘During the past 12 months, how often have you been so worried about something that you could not sleep at night?’. The answer options available to participants included ‘Never’, ‘Rarely’, ‘Sometimes’, ‘Most of the time’, and ‘Always’. We define responses of ‘Most of the time’ and ‘Always’ as stress-related sleep disturbance [16].

### Covariates

In our analyses, we included several covariates in our regression models due to their potential influence on stress-related sleep disturbances. These covariates were sex, age, having a close friend, tobacco use, alcohol use, marijuana use, fruit and vegetable intakes, purchasing power parity, and survey year.

### Statistical analysis

Due to the complex sampling design of the GSHS, we utilised primary sampling units, strata, and GSHS-based sampling weights to calculate the prevalence of sedentary behaviour, physical activity, and stress-related sleep disturbance in each country. The final sampling weight (W) was calculated as the product of the following components: W = W1 × W2 × f1 × f2 × f3, where W1 represents the inverse of the school selection probability, W2 denotes the inverse of the classroom selection probability within the school, f1 is a school-level non-response adjustment factor, f2 is a student-level non-response adjustment factor computed per classroom, and f3 is a post-stratification adjustment factor applied by sex within each grade. Based on the sample size of each country, sampling weights were recalculated to determine the overall prevalence of stress-related sleep disturbance as well as its prevalence across subgroups. Linear trend tests were used to assess the trends in the prevalence of stress-related sleep disturbances among adolescents with increasing sedentary time and physical activity time. We used multivariate logistic regression models to assess the odds ratio (OR) and 95% confidence interval (CI) of the association between sedentary behaviour and physical activity with stress-related sleep disturbance, after adjusting for potential covariates at the national level. All statistical analyses were performed using the “Complex Samples” module in SPSS 27.0 (International Business Machines Corporation, Armonk, New York, USA). A *P*-value <0.05 indicated statistically significance.

## RESULTS

### Participant characteristics

This study included 275 483 adolescents aged 12–17 years (boys = 50.9%) from 69 countries in five WHO regions (African = 10; American = 22; Eastern Mediterranean = 11; South East Asia = 8; Western Pacific = 18) in 2010–2019. The median sample size was 2322 and ranged from 106 in Tokelau to 53 557 in Argentina. Overall, the prevalence of sedentary behaviour was 30.0% and ranged from 10.4% in Nepal to 65.6% in Barbados. The prevalence of sufficient physical activity was 15.4% and ranged from 6.8% in Cambodia to 41.6% in Bangladesh. The prevalence of stress-related sleep disturbance was 8.3% and ranged from 3.5% in Myanmar to 22.9% in Afghanistan (Table S1 in the **Online Supplementary Document**).

### Proportion of stress-related sleep disturbance across different categories of sedentary time and physical activity

The overall proportion of stress-related sleep disturbance increased from 7.9% (95% CI = 7.5–8.3) to 15.6% (95% CI = 14.3–16.9), with increasing sedentary behaviour time (from the lowest <2 hours to the highest ≥7 hours) per day. Similar upward trends in proportion of stress-related sleep disturbance with increasing sedentary time were found in boys, girls, World Bank income categories, and five WHO regions (**Table 1**). The proportion of stress-related sleep disturbance shows a trend of first increasing and then decreasing with the number of days during the past seven days when engaged in physical activity for at least one hour per day. The same trend was observed in younger age groups (12–14 years), lower-middle-income countries, and regions of Eastern Mediterranean and Western Pacific (**Table 2**). In addition, the proportion of stress-related sleep disturbance was highest among adolescents with high sedentary behaviour and insufficient physical activity (12.1%, 95% CI = 11.4–12.8), followed by adolescents with high sedentary behaviour and sufficient physical activity (11.7%, 95% CI = 10.5–13.1), adolescents with low sedentary behaviour and insufficient physical activity (8.1%, 95% CI = 7.7–8.6), and adolescents with low sedentary behaviour and sufficient physical activity (6.6%, 95% CI = 5.7–7.6) (**Table 3**).

**Table 1:** Proportions (%) of stress-related sleep disturbance among adolescents by the time of sedentary behaviour*

Group	No. of countries	Sedentary behaviour time				*P* for trend
		**<2 h/d**	**3–4 h/d**	**5–6 h/d**	**≥7 h/d**	
**Total**	69	7.9 (7.5–8.3)	10.3 (9.6–11.1)	12.4 (11.2–13.7)	15.6 (14.3–16.9)	<0.001
**Sex**						
Males	69	6.9 (6.3–7.4)	8.7 (7.9–9.6)	10.0 (8.4–11.7)	12.9 (11.2–14.9)	<0.001
Females	69	9.0 (8.4–9.6)	12.1 (11.1–13.2)	15.1 (13.4–17.0)	18.2 (16.5–19.9)	<0.001
**Age group**						
12–14 y	69	6.3 (5.9–6.8)	9.0 (8.1–10.0)	11.7 (10.0–13.7)	13.3 (11.6–15.3)	<0.001
15–17 y	69	9.7 (9.1–10.5)	11.6 (10.4–12.9)	13.0 (11.4–14.8)	17.6 (15.8–19.4)	<0.001
**World Bank income group**						
Low income	8	8.3 (7.5–9.2)	11.5 (9.1–14.5)	14.3 (11.1–18.2)	14.6 (11.9–17.8)	<0.001
Lower-middle income	24	7.2 (6.7–7.7)	9.9 (9.0–10.9)	12.6 (11.0–14.5)	13.7 (11.8–15.8)	<0.001
Upper-middle income	19	9.4 (8.2–10.8)	10.3 (9.1–11.7)	10.8 (9.1–12.8)	17.9 (15.9–20.1)	<0.001
High income	18	10.6 (9.7–11.5)	12.2 (11.2–13.4)	15.6 (14.0–17.3)	21.8 (20.0–23.7)	<0.001
**WHO region**						
Africa	10	8.3 (7.0–9.7)	9.4 (7.9–11.2)	13.5 (10.7–16.9)	17.0 (14.0–20.5)	<0.001
Americas	22	8.6 (8.0–9.2)	10.4 (9.0-11.9)	12.8 (10.9–14.9)	16.5 (14.6–18.6)	<0.001
Eastern Mediterranean	11	6.7 (5.9–7.5)	10.0 (8.6–11.5)	12.0 (9.6–14.8)	14.5 (12.5–16.7)	<0.001
South-East Asia	8	4.2 (3.6–4.9)	5.5 (4.4–6.7)	10.5 (8.3–13.2)	12.0 (9.6–15.0)	<0.001
Western Pacific	18	9.5 (8.7–10.4)	12.5 (11.1–14.2)	12.9 (10.5–15.7)	16.1 (13.0–19.7)	<0.001

CI – confidence interval, WHO – World Health Organization

*Data is presented as % (95% CI).

**Table 2:** Proportions (%) of stress-related sleep disturbance among adolescents by the days of physical activity ≥** **1 hour/day during the past seven days*

Group	No. of countries	Days of physical activity ≥1 h/d during the past 7 d					*P* for trend
		**0 d**	**1–2 d**	**3–4 d**	**5–6 d**	**7 d**	
**Total**	69	9.1 (8.6–9.6)	9.4 (8.9–10.0)	9.5 (8.8–10.2)	8.4 (7.2–9.7)	8.0 (7.2–8.9)	0.009
**Sex**							
Males	69	7.9 (7.2–8.7)	8.2 (7.6–9.0)	7.9 (7.0–9.0)	6.9 (5.3–8.8)	6.8 (5.8–7.9)	0.081
Females	69	10.2 (9.5–10.9)	10.5 (9.8–11.3)	11.5 (10.3–12.7)	10.6 (8.9–12.6)	10.0 (8.8–11.3)	0.331
**Age group**							
12–14 y	69	7.4 (6.8–8.1)	8.0 (7.3–8.7)	7.5 (6.7–8.4)	6.7 (5.5–8.1)	6.3 (5.4–7.3)	0.024
15–17 y	69	11.3 (10.5–12.1)	10.9 (10.2–11.8)	11.5 (10.4–12.7)	10.0 (8.3–12.0)	9.9 (8.7–11.1)	0.137
**World Bank income group**							
Low income	8	9.1 (8.0–10.2)	9.5 (8.4–10.7)	9.7 (8.0–11.6)	8.5 (6.7–10.8)	9.6 (7.9–11.7)	0.819
Lower-middle income	24	8.4 (7.7–9.1)	9.1 (8.4–9.9)	8.6 (7.7–9.6)	7.4 (5.8–9.5)	6.2 (5.3–7.3)	<0.001
Upper-middle income	19	11.0 (9.5–12.6)	9.7 (8.7–10.8)	10.8 (9.3–12.6)	10.1 (8.3–12.2)	11.9 (10.1–14.0)	0.259
High income	18	14.3 (13.0–15.7)	13.2 (12.1–14.4)	13.2 (11.7–14.9)	12.8 (11.1–14.8)	14.6 (13.3–16.1)	0.135
**WHO region**							
Africa	10	9.3 (7.6–11.4)	9.3 (7.9–11.1)	8.5 (7.0–10.4)	5.6 (3.9–8.0)	12.3 (10.2–14.8)	<0.001
Americas	22	9.8 (8.9–10.7)	9.5 (8.8–10.4)	10.0 (8.9–11.3)	9.3 (8.0–10.9)	10.2 (8.9–11.5)	0.837
Eastern Mediterranean	11	8.0 (7.0–9.1)	8.8 (7.6–10.1)	9.2 (7.9–10.7)	6.8 (5.1–9.2)	5.9 (4.7–7.4)	0.005
South-East Asia	8	5.3 (4.4–6.3)	4.8 (4.2–5.6)	5.2 (4.0–6.7)	5.7 (4.2–7.8)	6.3 (5.0–7.7)	0.427
Western Pacific	18	10.6 (9.5–11.7)	11.5 (10.4–12.7)	10.7 (9.3–12.3)	10.5 (7.6–14.4)	8.7 (7.1–10.7)	0.105

CI – confidence interval, WHO – World Health Organization

*Data is presented as % (95% CI).

**Table 3:** Proportions (%) of stress-related sleep disturbance among adolescents by combined categories of sedentary behaviour and physical activity*

Group	No. of countries	Low sedentary behaviour		High sedentary behaviour	
		**Sufficient physical activity**	**Insufficient physical activity**	**Insufficient physical activity**	**Sufficient physical activity**
**Total**	69	6.6 (5.7–7.6)	8.1 (7.7–8.6)	12.1 (11.4–12.8)	11.7 (10.5–13.1)
**Sex**					
Males	69	5.5 (4.5–6.7)	7.2 (6.6–7.8)	9.9 (9.2–10.7)	10.0 (8.3–12.1)
Females	69	8.3 (7.0–9.9)	9.1 (8.5–9.7)	14.2 (13.2–15.1)	14.6 (12.7–16.8)
**Age group**					
12–14 y	69	4.8 (3.9–5.9)	6.7 (6.2–7.1)	10.5 (9.7–11.4)	10.7 (8.8–12.9)
15–17 y	69	8.7 (7.3–10.2)	10.0 (9.2–10.7)	13.4 (12.5–14.4)	12.7 (10.9–14.8)
**World Bank income group**					
Low income	8	8.1 (6.4–10.3)	8.4 (7.6–9.2)	12.5 (10.6–14.8)	14.2 (10.9–18.3)
Lower-middle income	24	5.1 (4.1–6.2)	7.7 (7.1–8.3)	11.5 (10.7–12.5)	10.0 (8.3–12.0)
Upper-middle income	19	11.1 (8.8–13.9)	9.2 (7.9–10.6)	12.2 (11.2–13.4)	13.2 (11.0–15.8)
High income	18	12.3 (10.7–14.2)	10.2 (9.3–11.3)	15.9 (14.8–17.1)	16.7 (14.9–18.7)
**WHO region**					
Africa	10	11.2 (8.7–14.5)	7.9 (6.5–9.4)	11.5 (10.0–13.2)	14.0 (10.8–18.0)
Americas	22	8.8 (7.6–10.3)	8.6 (7.9–9.2)	12.3 (11.3–13.5)	13.0 (10.8–15.6)
Eastern Mediterranean	11	4.6 (3.4–6.3)	7.3 (6.5–8.2)	11.6 (10.4–13.0)	10.7 (8.5–13.4)
South-East Asia	8	4.5 (3.1–6.5)	4.2 (3.6–4.8)	7.6 (6.6–8.8)	10.0 (7.7–12.7)
Western Pacific	18	7.6 (5.9–9.7)	9.9 (9.0–10.8)	13.9 (12.3–15.6)	11.4 (8.9–14.5)

CI – confidence interval, WHO – World Health Organization

*Data is presented as % (95% CI)

### The association of stress-related sleep disturbance with sedentary behaviour and physical activity

**Figure 1:**
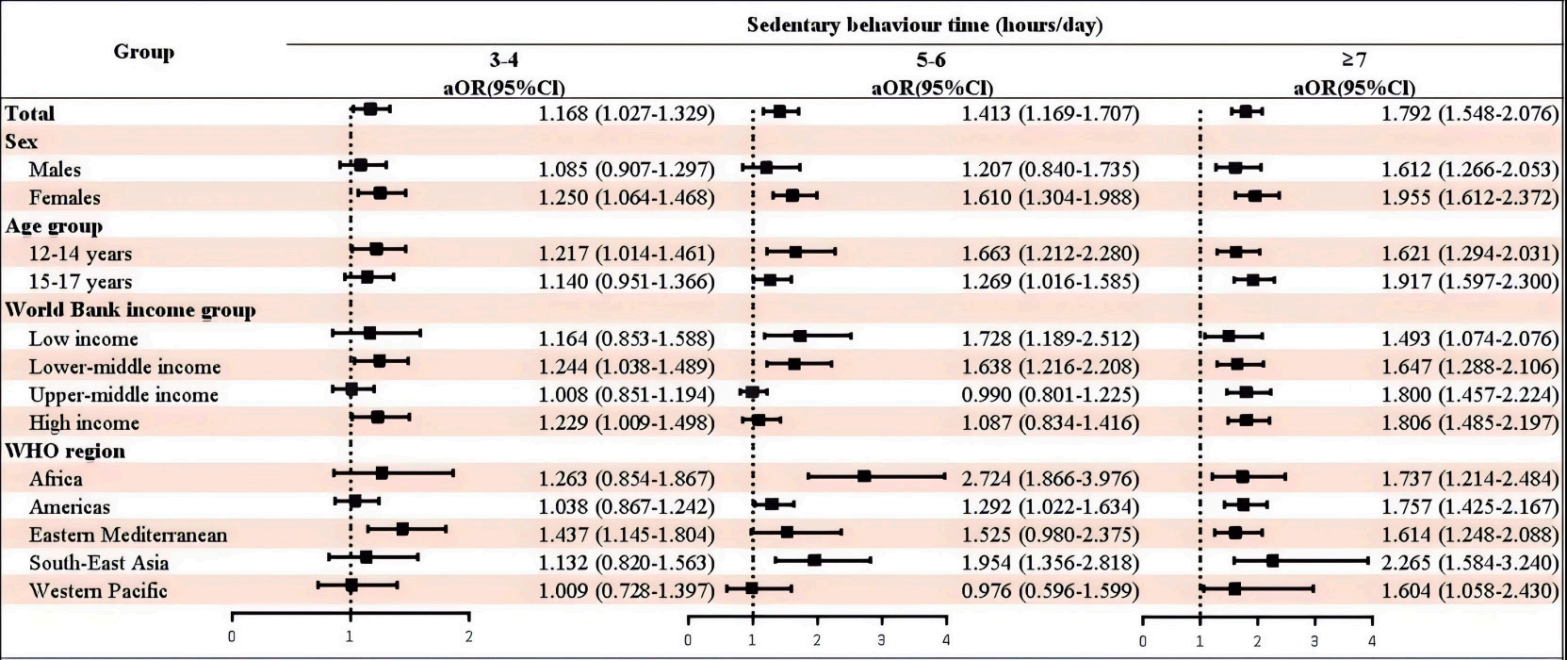
Association between sedentary behaviour and stress-related sleep disturbance among adolescents by sex, age group, World Bank income group, WHO region. Reference group: sedentary time less than 2 hour/day. CI – confidence interval, OR – odds ratio, WHO – World Health Organization.

**Figure 2:**
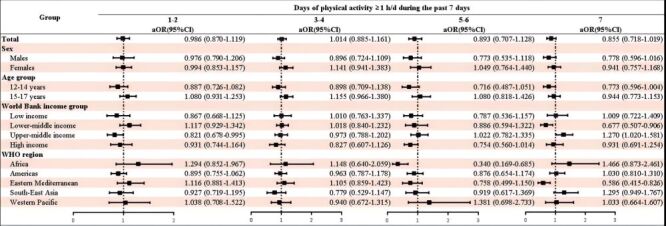
Association between physical activity and stress-related sleep disturbance among adolescents by sex, age group, World Bank income group, WHO region. Reference group: 0 days of physical activity ≥ 1 hour/day during the past seven days). CI – confidence interval, OR – odds ratio, WHO – World Health Organization.

**Figure 3:**
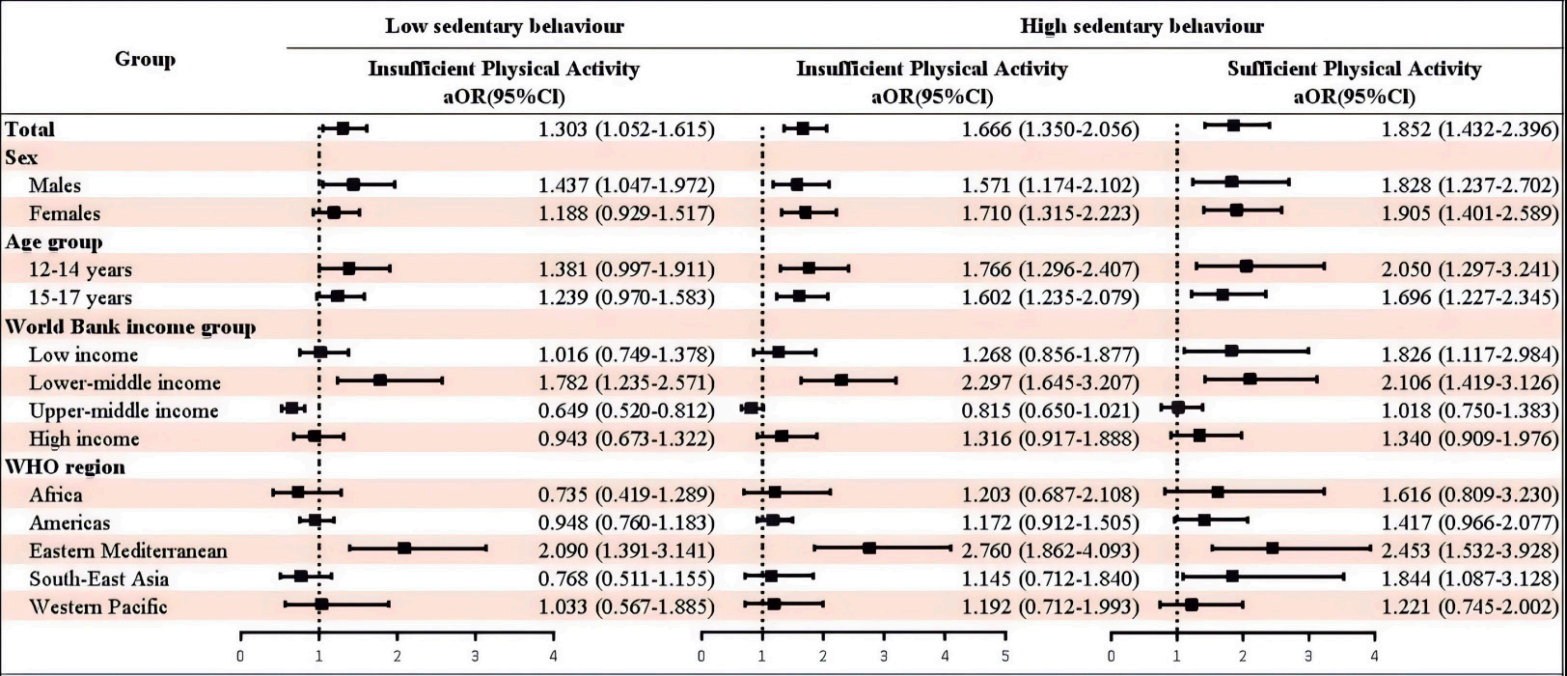
Association of sedentary behaviour alone, physical activity alone, or combined performance of both behaviour with stress-related sleep disturbance among adolescents by sex, age group, World Bank income group, WHO region. Reference group: adolescents with low sedentary behaviour and sufficient physical activity). CI – confidence interval, OR – odds ratio, WHO – World Health Organization.

Compared to adolescents with sedentary time less than 2 hours/d, increased sedentary time was positively associated with stress-related sleep disturbance: 3–4 hours/d (OR = 1.168; 95% CI = 1.027–1.329); 5-6 hours/d (OR = 1.413; 95% CI = 1.169–1.707); ≥7 hours/d (OR = 1.792; 95% CI = 1.548–2.076) after adjusting for potential confounding covariates. Subgroup analyses by sex, age group, World Bank income group, and WHO region showed similar results (**Figure 1**). Although the decrease in the risk of stress-related sleep disturbance in adolescents with increased days of engaged physical activity at least 1 hour per day during the past seven days was not statistically significant, a protective trend was observed (**Figure 2**). After adjusting for potential confounding covariates, compared with adolescents with low sedentary behaviour and sufficient physical activity, adolescents with low sedentary behaviour and insufficient physical activity (OR = 1.303; 95% CI = 1.052–1.615), high sedentary behaviour and insufficient physical activity (OR = 1.666; 95% CI = 1.350–2.056), and adolescents with high sedentary behaviour and sufficient physical activity (OR = 1.852; 95% CI = 1.432–2.396) were positively associated with stress-related sleep disturbance. Subgroup analyses by sex, age group, World Bank income group, and WHO region showed similar results (**Figure 3**).

## DISCUSSION

Our study summarises the updated GSHS data (from 2010 to 2019) for adolescents aged 12–17 years in 69 countries. We found a relatively low prevalence of sufficient physical activity, a high prevalence of sedentary behaviour, and a notable prevalence of stress-related sleep disturbance. Importantly, our study reveals a significant ‘dose-response’ relationship between sedentary behaviour and stress-related sleep disturbances, which remained consistent and robust across all subgroup analyses stratified by gender, age group, World Bank income group, and WHO region. In contrast, the association between physical activity and stress-related sleep disturbances demonstrated a more complex and ambiguous pattern in relation to stress-related sleep disturbance.

Our findings suggest that adolescents aged 15–17 and those in high-income countries show a higher prevalence of stress-related sleep disturbance. The prevalence of stress-related sleep disturbance increases with more sedentary time compared to <2 hours/d. A recent data analysis found that 59% of adolescents in the USA spend at least two hours per day sitting and watching television or videos [17]. This is likely related to the level of economic development, where adolescents tend to stay at home watching television and playing computer games, leading to a lack of physical activity [18], which aligns with our findings. A study found that increase sedentary time is associated with a higher risk of cardiovascular disease and all-cause mortality [19].

Previous study found that the prevalence of sufficient activity was low among adolescents worldwide [20]. This aligns with our earlier observations, which showed that only 15.4% of adolescents engage in sufficient physical activity. A cross-sectional survey conducted in 2016 across 146 countries revealed that 81% of adolescents aged 11–17 years had insufficient physical activity levels [21]. However, in our analysis, the association between physical activity and stress-related sleep disturbances was not statistically significant and demonstrated a nonlinear pattern. This relationship may not follow a simple linear trajectory and is likely obscured by the stronger association with sedentary behaviour observed in our study. The observed high sedentary behaviour may partially stem from academic pressures and excessive reliance on screen-based entertainment, which coexists with physical inactivity to form a cluster of detrimental health behaviours [22,23]. Therefore, our findings suggest that intervention efforts could consider placing a strong emphasis on reducing adolescent sedentary behaviour, with specific emphasis on implementing standardised screen time restrictions and developing coordinated environmental restructuring strategies between homes and schools.

We also found that adolescents with both high sedentary behaviour and sufficient physical activity had elevated odds of stress-related sleep disturbance. Notably, this risk was not lower than that observed in peers with high sedentary behaviour but insufficient physical activity. The observed sleep phenomena can be attributed to the synergistic effects of multiple factors. Prolonged screen exposure directly impacts sleep by reducing duration and delaying onset, whereas acute bouts of high-intensity exercise may transiently disrupt sleep through excessive sympathetic activation [24,25]. Furthermore, prolonged sedentary behaviour, particularly when accompanied by evening screen exposure, not only suppresses endogenous melatonin secretion and delays circadian phase but also interacts with chronic psychological stress to induce sustained sympathetic activation [8,26,27]. Those who meet sufficient physical activity may experience a 'licensing effect', mentally permitting themselves compensatory increases in recreational sedentary behaviour. Concurrently high sedentary time (potentially due to academic pressure) and mandatory/intense physical activity may represent behavioural manifestations of unmeasured latent stressors, such as high-achievement anxiety or parental pressure [22,28,29]. Moreover, the timing and pattern of physical activity also play significant roles. Vigorous evening exercise may acutely disrupt sleep via sympathetic nervous system activation, potentially exacerbating sleep disturbances associated with high sedentary time [30]. Therefore, explicit guidelines regarding physical activity intensity, duration, and timing are needed. Beyond promoting regular moderate-intensity activity, public health initiatives should also explicitly address the reduction of recreational sedentary behaviour, especially in the pre-sleep period, as crucial for protecting adolescent sleep.

We found that the prevalence of stress-related sleep disturbances is higher in girls than in boys. This may be related to physiological factors due to gender differences, as a former study showed that boys have higher levels of testosterone than girls, which enables them to benefit from greater strength and endurance during physical activity [31]. Another previous study also showed that boys had a higher percentage of effective physical activity and were more active than girls [32]. Even more than that, Sales et al. found that school environment indexes were associated with higher levels of physical activity [33]. These findings highlight the urgency of introducing and implementing policies and interventions that encourage more exercise, particularly among females, by improving the physical activity environment and increasing the number of sports facilities.

Research indicates that physical activity offers numerous benefits, such as enhancing cardiovascular health, reducing the incidence of diseases, and promoting the growth and development of adolescents [34]. Additionally, evidence suggests that increasing physical activity can effectively lower the prevalence of various diseases, and improve overall health [35]. However, our study has found that even when the recommended daily amount of physical activity is achieved, the risk of stress-related sleep disturbances associated with sedentary behaviour cannot be entirely eliminated. Therefore, we recommend combined intervention strategies that explicitly target a reduction in recreational sedentary behaviour as a primary objective, alongside the promotion of physical activity for its overarching health benefits. Public health initiatives should provide clear guidance on limiting screen time and other sedentary pursuits, ensuring adolescents have adequate opportunities for active leisure, and raising awareness that moving more must be coupled with sitting less to fully benefit sleep health.

### Strengths and limitations

This study has several strengths. First, it is based on the most recent GSHS data (2010–2019), which included a total of 275 483 adolescents from multiple countries and regions around the world. This large and diverse sample was used to assess the association between sedentary behaviour and physical activity with stress-related sleep disturbance, greatly enhancing the generalisability of the study’s results. Second, the same sampling method was employed to ensure the rigor of the data collection, and the same questionnaire was used in each country to guarantee the comparability of the data. However, there are also some limitations in our study. First, given the cross-sectional nature of this study, the temporal sequence and causal inference between exposures and the outcome cannot be established. Although multiple potential confounders were adjusted for in our analyses, the possibility of residual confounding or reverse causality remains. Future prospective studies or designs employing more rigorous causal inference methods (*e.g*. instrumental variable analysis, cross-lagged models) will be essential for verifying the directionality of these associations. Second, the dichotomisation of self-reported behaviours based on standard guidelines, while pragmatic for surveillance, may introduce non-differential misclassification. This is particularly relevant for our definitions: ‘high sedentary behaviour’ (≥ 2 hours/day) follows a public health guideline but may not capture relevant biological thresholds, and ‘sufficient physical activity’ (defined as meeting the ≥60 minute/day threshold on all seven recalled days) represents a stricter interpretation of the WHO recommendation (an average of 60 minute/day across the week). Such categorisation cannot capture behavioural spectra (*e.g*. patterns of sedentary accumulation or physical activity intensity), potentially attenuating the observed effect sizes. Future studies would benefit from using device-based measures (*e.g*. accelerometers) to objectively quantify behaviours with greater precision, enabling analyses that test both guideline-based thresholds and continuous, data-driven exposures. Third, the temporal reference periods differed across variables, this mismatch may affect the interpretation of temporal relationships between exposures and outcome. Fourth, the use of a single questionnaire item for stress-related sleep disturbance precludes the examination of potential differential associations with more specific subtypes (*e.g*. anxiety-related insomnia *vs*. sleep-onset difficulty). Furthermore, residual confounding by factors such as underlying mental health status or chronic stress cannot be fully ruled out despite covariate adjustment, introducing some uncertainty to the observed associations. Future research should employ multidimensional sleep instruments alongside detailed psychosocial assessments to better delineate behavioural relationships with specific sleep phenotypes. Fifth, due to limitations in the GSHS data, we could not determine which sedentary behaviours or physical activities have the greatest impact on stress-related sleep disturbance. Therefore, further research is necessary to address these limitations.

## CONCLUSIONS

Our study shows relatively high prevalence of sedentary behaviour and relatively low prevalence of sufficient physical activity among adolescents aged 12–17 years. Sedentary behaviour is positively correlated with stress-related sleep disturbance. In conclusion, our study provides evidence of a consistent association between sedentary behaviour and a higher prevalence of stress-related sleep disturbance among adolescents. Although a trend suggested a potential benefit of higher physical activity levels, this association may not represent an independent protective effect. Specifically, sedentary behaviour and physical activity vary by gender, age, income, and WHO region. Importantly, achieving sufficient physical activity does not confer protection against the stress-related sleep disturbance effects of high sedentary time. Therefore, public health initiatives should consider prioritising the reduction of recreational sedentary behaviour as a key strategy for improving adolescent stress-related sleep disturbances. Concurrently, continued promotion of physical activity remains essential for its broader health benefits. Additionally, support from all sectors of society is needed to increase adolescent’s physical activity, reduce sedentary behaviour, ensure adolescents’ sleep quality, and promote their physical health. Our findings provide critical preliminary evidence and specific targets for future longitudinal or experimental research testing causal hypotheses.

## Additional material


Online Supplementary Document
