# Supplementary material for: Associations of sedentary behaviour and physical activity with stress-related sleep disturbance among adolescents in 69 countries: a population-based study
Source: J Glob Health. 2026 Feb 6;16:04049. doi: 10.7189/jogh.16.04049 (PMC12879260; doi:10.7189/jogh.16.04049)
Supplement: Online Supplementary Document [file jogh-16-04049-s001.pdf]

Supplement to: Li J, Zhu Y, Huang D, Pan M, Li F, Li L, Sun J, Ma C, Zhang B. Associations of sedentary behaviour and physical activity with stress-related sleep disturbance among adolescents in 69 countries: A population-based study. J Glob Health. 2026;16:04049.

Table S1. Characteristics of adolescents aged 12-17 years in 69 countries, 2010-2019

Country	Survey year	Sample size	Mean age, years	Boy, %	Sedentary behaviour (≥2h/d), %	Sufficient physical activity (≥1h/d), %	Sleep Disturbance, %
**Africa**							
Benin	2016	1549	15.5	69.0	25.1	29.3	18.2
Ghana	2012	2322	15.1	50.2	19.6	11.9	13.2
Liberia	2017	1067	15.4	52.3	20.8	11.2	16.8
Mauritania	2010	1845	14.8	54.3	37.4	12.5	11.4
Mauritius	2017	2807	14.9	46.3	40.4	19.0	9.1
Mozambique	2015	1224	15.4	52.5	38.6	12.9	9.4
Namibia	2013	3195	15.1	44.4	36.3	14.0	14.1
Seychelles	2015	2309	13.9	48.7	51.2	17.6	11.0
Sierra Leone	2017	2306	15.0	50.1	24.2	18.7	17.7
Tanzania	2014	3230	14.3	49.1	19.9	20.3	5.7
**America**							
Anguilla	2016	752	14.7	48.2	60.0	19.0	9.9
Argentina	2018	53,557	14.9	47.8	55.4	16.4	13.3
Bahamas	2013	1234	13.4	47.2	54.9	15.4	13.7
Barbados	2011	1503	14.2	48.5	65.6	18.6	10.0

Belize	2011	1827	14.0	48.1	38.0	21.2	12.4
Bolivia	2018	6242	15.2	50.2	31.4	11.5	13.0
Curacao	2015	2011	14.7	48.1	61.3	11.8	10.8
Dominican Republic	2016	1257	14.7	49.8	45.6	13.1	10.1
El Salvador	2013	1823	14.3	51.8	34.7	12.9	6.9
Guatemala	2015	3756	14.3	52.6	22.3	11.1	6.5
Guyana	2010	2259	14.4	48.6	36.1	15.6	14.0
Honduras	2012	1647	14.0	46.9	30.2	15.9	5.6
Jamaica	2017	1490	15.0	47.5	56.8	22.8	13.2
Panama	2018	2531	15.1	46.9	48.6	14.7	10.0
Paraguay	2017	2814	14.7	48.4	34.8	17.0	9.0
Peru	2010	2806	14.5	50.4	28.8	15.2	8.9
Saint Kitts and Nevi	2011	1656	14.4	43.6	59.7	18.1	8.9
Saint Lucia	2018	1762	14.4	47.2	56.1	21.0	14.1
Saint Vincent and the Grenadines	2018	1630	15.1	47.7	55.3	18.3	15.8
Suriname	2016	1850	14.5	49.0	43.9	19.1	12.4
Trinidad and Tobago	2017	3370	14.2	47.5	49.3	20.2	13.8
Uruguay	2012	3344	14.4	45.4	59.3	15.9	5.8
**Eastern Mediterranean**							
Afghanistan	2014	2044	14.8	53.7	24.6	9.5	22.9

Bahrain	2016	6784	14.2	50.5	59.0	19.9	16.3
Iraq	2012	1862	14.4	57.0	26.4	14.9	13.0
Kuwait	2015	2916	15.0	50.2	65.4	15.8	20.2
Lebanon	2017	4769	14.7	45.5	44.9	14.6	13.5
Morocco	2016	5426	14.5	52.7	31.2	10.7	15.9
Palestine	2010	13,457	13.9	48.7	32.8	16.5	16.3
Qatar	2011	1526	13.5	47.2	48.1	10.7	18.2
Syria	2010	2929	13.7	51.5	25.4	11.3	14.7
United Arab Emirates	2016	5187	14.8	47.6	59.3	15.8	15.9
Yemen	2014	2100	14.6	53.4	21.6	12.3	14.6
**South East Asia**							
Bangladesh	2014	2816	14.2	65.4	15.2	41.6	4.2
Bhutan	2016	5976	15.1	46.1	29.6	14.6	7.6
Indonesia	2015	10,338	14.0	48.9	27.3	12.2	4.5
Myanmar	2016	2692	14.2	46.2	15.9	10.8	3.5
Nepal	2015	5985	14.5	49.4	10.4	15.5	4.2
Sri Lanka	2016	3143	14.7	48.3	37.3	15.5	4.6
Thailand	2015	5399	14.4	45.7	56.5	11.4	8.5
Timor-Leste	2015	2821	15.2	48.1	14.8	9.5	11.3
**Western Pacific**							
Brunei Darussalam	2019	2251	14.6	50.4	56.2	11.8	15.0

Cambodia	2013	2861	15.0	50.0	11.7	6.8	5.0
Cook Islands	2015	643	15.2	48.6	45.0	15.5	13.9
Fiji	2016	2843	15.4	48.5	28.1	20.6	12.0
French Polynesia	2015	2733	14.8	49.4	40.7	17.7	11.5
Kiribati	2011	1528	14.3	46.8	15.1	17.7	9.1
Laos	2015	3566	15.6	53.0	20.9	16.7	4.8
Malaysia	2012	24,939	14.9	49.9	47.4	14.2	5.3
Mongolia	2013	5026	14.4	48.2	44.4	25.2	5.5
Nauru	2011	462	14.5	39.6	40.0	13.4	16.9
Philippines	2015	8137	14.5	48.5	31.6	7.6	10.7
Samoa	2017	1537	14.9	45.9	27.0	22.3	9.4
Solomon Islands	2011	1217	14.7	54.5	25.5	16.5	12.7
Tokelau	2014	106	14.2	56.3	51.4	25.4	10.4
Tonga	2017	2756	14.4	50.4	21.2	17.8	14.4
Tuvalu	2013	836	14.0	48.1	17.1	11.6	6.2
Vanuatu	2016	1958	14.9	49.0	20.7	12.6	6.7
Wallis and Futuna	2015	939	14.5	47.8	36.9	13.4	15.8
**Total**		275,483	14.7	50.9	30.0	15.4	8.3
